# Valve disease and aortopathy associations of bicuspid aortic valve phenotypes differ between men and women

**DOI:** 10.1136/openhrt-2021-001857

**Published:** 2021-10-20

**Authors:** Carl Granath, Salah A Mohamed, Christian Olsson, Michael Grattan, Luc Mertens, Anders Franco-Cereceda, Hanna M Björck

**Affiliations:** 1 Department of Molecular Medicine and Surgery, Section of Cardiothoracic Surgery, Karolinska Institutet, Stockholm, Sweden; 2 Department of Cardiac and Thoracic Vascular Surgery, Universitätsklinikum Schleswig-Holstein Campus Lübeck, Lubeck, Germany; 3 Department of Thoracic and Cardiovascular Surgery, Karolinska University Hospital, Stockholm, Sweden; 4 Department of Paediatrics, LHSC Children's Hospital, University of Western Ontario, London, Ontario, Canada; 5 Department of Paediatrics, Division of Cardiology, The Hospital for Sick Children, University of Toronto, Toronto, Ontario, Canada; 6 Department of Medicine, Cardiovascular Medicine Unit, Center for Molecular Medicine, Karolinska Institutet, Karolinska University Hospital, Stockholm, Sweden

**Keywords:** aortic aneurysm, epidemiology, aortic valve insufficiency, aortic valve stenosis, heart defects, congenital

## Abstract

**Objective:**

Determine whether associations between bicuspid aortic valve (BAV) phenotypes, valve disease and aortopathy differ between sexes.

**Methods:**

1045 patients with BAV (76.0% men, n=794) from two surgical centres were included in this cross-sectional study. Valve phenotype was classified intraoperatively as right–left (RL), right-non-coronary (RN), left-non-coronary (LN) or 2-sinus BAV. Echocardiography was used to determine type and degree of valve disease, and aortic dimensions. Aortic dilatation was defined as diameter ≥4.5 cm.

**Results:**

RL was the most common phenotype (73.6%), followed by RN (16.2%), 2-sinus BAV (9.2%) and LN (1.1%), with no difference in phenotype distribution between men and women (p=0.634). Aortic valve insufficiency (AI) prevalence differed significantly with valve phenotype in men (p=0.047), with RL and LN having the highest prevalence (34.1% and 44.0%, respectively). In women, RN had a higher proportion of AI than RL (21.3% vs 7.3%, p=0.017). Men with RL had larger root dimensions, in particular at the sinus (mean difference 0.24 cm compared with RN, p=0.002). Men with 2-sinus BAV had the highest prevalence of root phenotype dilatation (7.0%, other phenotypes ≤2.3%, p=0.031), whereas women with 2-sinus BAV did not have root dilatation and smaller sinus dimensions (mean difference: 0.35 cm compared with RL, p=0.021). Aortic root segments were larger in men with AI compared with aortic stenosis (sinus mean difference: 0.40 cm, p<0.001). The difference was even larger in women (mean difference: 0.78 cm, p<0.001), and women with AI also had larger tubular segments (mean difference: 0.61 cm, p=0.001).

**Conclusions:**

There are significant sex differences in clinical associations of BAV phenotypes, which should be considered in further studies on the role of phenotypes in individualised patient management.

**Key questions**

**What is already known on this subject?**

- Bicuspid aortic valve (BAV) is frequently associated with aortic valve disease and/or ascending aortic aneurysm. The BAV morphology has been shown to correlate with type of valve disease and aortic dilatation, mainly in cohorts selected through echocardiography. Specifically, right–left phenotype (RL) has been associated with larger aortic root dimensions and/or root dilatation, right-non-coronary (RN) phenotype has been associated with a higher prevalence of aortic valve stenosis, and 2-sinus BAV may be associated with root phenotype aortic dilatation.

**What might this study add?**

- The present study is one of the largest surgical cohorts of patients with BAV, which provides definitive BAV diagnosis and phenotype classification. The results show that associations between BAV phenotypes, valve disease, aortic dimensions and aortopathy are sex dependent, which has not been described before. Specifically, men with RL and left-non-coronary phenotype had a higher prevalence of aortic valve insufficiency (AI), while RN had the highest prevalence of AI in women. Further, 2-sinus BAV was strongly associated with root phenotype aortic dilatation in men, while women with 2-sinus BAV did not have root dilatation and even had smaller root dimensions than other phenotypes. Differences in aortic dimensions and dilatation prevalence between aortic stenosis and AI were greater in women than in men. The study also verifies some previous findings in non-surgical cohorts, such as the association between larger root dimensions and RL, which was seen in male patients.

**How might this impact on clinical practice?**

- The finding that the clinical impact of BAV phenotypes is sex dependent may lead to improved individualised decision-making regarding diagnostic and therapeutic options. This is particularly important as transcatheter aortic valve replacement indications may expand to include more patients with BAV.

## Introduction

Bicuspid aortic valve (BAV) is the most common congenital cardiac malformation, with an estimated prevalence of ~1% in the population, and a roughly three times higher prevalence in men.^1–4^ It is associated with a dramatically increased risk of both aortic valve disease and ascending aortic aneurysm.^4 5^ Several morphologically distinct BAV phenotypes exist, categorised according to the leaflet fusion pattern, presence or absence of a raphe and number of sinuses.^6 7^ These phenotypes result in different flow patterns in the ascending aorta,^8 9^ and preclinical studies suggest that they are caused by different developmental abnormalities.^10^ Some studies in adults have found correlations between right–left (RL) fusion and larger aortic root and/or root dilatation,^11–14^ and a single study has shown a correlation between right-non-coronary (RN) fusion and aortic valve stenosis (AS).^13^ Others have found that RL fusion may have more aortic dilatation overall, whereas 2-sinus BAV, with two equally sized leaflets and no raphe, was associated with a lower prevalence of significant valve disease and aortic dilatation.^15^ However, most cohorts investigating the clinical significance of BAV phenotypes have been selected through echocardiography, which has a limited sensitivity for BAV diagnosis and an uncertain diagnostic accuracy for phenotype classification.^16^ Surgical inspection is considered to provide definitive BAV diagnosis and phenotype classification, and is used as a reference for assessment of other diagnostic modalities,^16 17^ and the largest surgical cohort to date found only a weak correlation between 2-sinus BAV and aortic root dilatation.^18^ The clinical impact of the BAV phenotype thus remains controversial. Further, while sex differences in BAV have been increasingly recognised,^3 19 20^ no study to date has investigated whether BAV phenotype associations differ between sexes. A better understanding of associations between sex and BAV phenotype is important to inform diagnostic and therapeutic strategies, including the optimal timing of surgical treatment, and is particularly important as patients with BAV are increasingly considered for transcatheter aortic valve replacement.^21^

The present study evaluates the clinical implications of BAV phenotypes in men and women, respectively, in one of the largest surgical BAV cohorts. The primary aim was to determine the impact of BAV phenotype on the type of valve disease, aortic dimensions and the prevalence and type of aortopathy, in men and women. A secondary aim was to study the relationship between the type of valve disease and aortic dimensions after stratification by sex.

## Material and methods

### Patient characteristics

A two-centre, cross-sectional cohort study of adult patients with BAV with aortic valve disease and/or ascending aortic dilatation was conducted. A total of 1291 patients aged ≥18 years at the time of echocardiography who had undergone primary open aortic valve surgery or aortic root surgery, with or without concomitant repair of the ascending aorta or other cardiac procedures, at two adult cardiac surgical centres (Karolinska University Hospital, Sweden and University Medical Center Schleswig-Holstein Campus Luebeck, Germany) were included between September 1995 and September 2015. Exclusion criteria were genetic syndromes and diseases (n=12), history of endocarditis (n=6), surgery for subvalvular aortic stenosis (n=55), systemic inflammatory disease or vasculitis (n=4) and aortic dissection (n=1). Additionally, 168 patients were excluded due to an indeterminate, missing or unicuspid valve phenotype.

### Technical information

All patients underwent transthoracic echocardiography prior to surgery at the respective institution to determine aortic valve dysfunction. The mean pressure gradient was used to determine severity of stenosis, which was classified as mild (<20 mm Hg), moderate (20–39.9 mm Hg) or severe (≥40 mm Hg). Alternatively, if there was no documented gradient, AS severity was classified by the sonographer (n=27). Aortic valve insufficiency (AI) was classified as mild (grade 1), moderate (grade 2) or severe (grade 3–4) by the sonographer. Valve disease was considered significant if moderate or severe (AI grade ≥2, mean gradient ≥20 mm Hg), with AS being considered the primary lesion in patients with mixed valve disease. Patients were subsequently classified as having either AS, AI or dilatation only. Cases with incomplete data (n=13) were categorised based on the primary surgical indication. The dominant valve lesion was used to determine surgical indication, with stenosis being considered dominant in cases with mixed valve disease of equal severity.

Aortic dimensions were measured with either preoperative transthoracic echocardiography or intraoperative transesophageal echocardiography. The maximum diameter was measured at the level of the aortic valve annulus, sinus of Valsalva, sinotubular junction and the tubular segment of the ascending aorta. The annulus was measured from inner edge to inner edge at midsystole, all other dimensions were measured from leading edge to leading edge at end diastole. Based on current guidelines for aortic intervention in patients with BAV, aortopathy was defined as any aortic segment (sinus, sinotubular junction or tubular ascending aorta) ≥4.5 cm in diameter.^22^ Aortic root dilatation was defined as sinus of Valsalva and/or sinotubular junction diameter ≥4.5 cm, and ascending aortic dilatation as tubular segment diameter ≥4.5 cm. Aortic dilatation pattern was subsequently classified as root phenotype (root dilatation only), ascending phenotype (tubular segment dilatation only) or root extended phenotype (dilatation of both the root and tubular segment).^7^ The BAV phenotype was determined intraoperatively through direct visual inspection by the surgeon. After assessment of the presence or absence of raphe, the number of sinuses and commissures and their morphology, the phenotype was classified as right–left (RL) fusion, right-non-coronary (RN) fusion, left-non-coronary (LN) fusion or as 2-sinus BAV if two equally sized cusps without a raphe, and two sinuses and commissures, were observed.

The medical records were reviewed at the respective institution to find information on coexistent cardiac pathology, genetic syndromes and other conditions that may affect the aortic valve or thoracic aorta, as well as cardiovascular risk factors. Hypertension was defined as a diagnosis of hypertension in the medical records and treatment with any class of antihypertensive medication. Dyslipidaemia was similarly defined as recorded diagnosis of hypercholesterolaemia and treatment with any lipid-lowering medication. At the time of echocardiography, the patient’s height and weight were recorded, and body surface area (BSA) was calculated using the Mosteller formula.

### Patient and public involvement

Patients or the public were not involved in the design, conduct, reporting, or dissemination plans of the research.

### Statistics

Continuous variables are presented as mean±SD. Nominal variables were summarised using frequencies and proportions. Between-group differences in continuous variables were assessed with independent samples t-test, assuming equal or unequal variance as appropriate, or one-way analysis of variance with Tukey honestly significant difference post hoc analysis. If the n-numbers in all groups were less than 100 and the data were significantly skewed, a Mann-Whitney U-test was used instead to assess differences in continuous variables, with the data presented as median (IQR). Analysis of covariance was used to adjust for age and BSA in comparisons of continuous variables between groups, with Bonferroni corrections if multiple groups. Differences between groups in nominal variables were assessed with χ^2^ test, or Fisher’s exact test when appropriate, with Bonferroni corrections. Binary logistic regression was used for multivariate analysis, with data presented as OR. Cases with missing data were excluded on an analysis-by-analysis basis. All statistical analyses were performed in SPSS, V.26.0 (IBM). A two-sided p value<0.05 was considered significant.

## Results

### Patient characteristics

**Figure 1:**
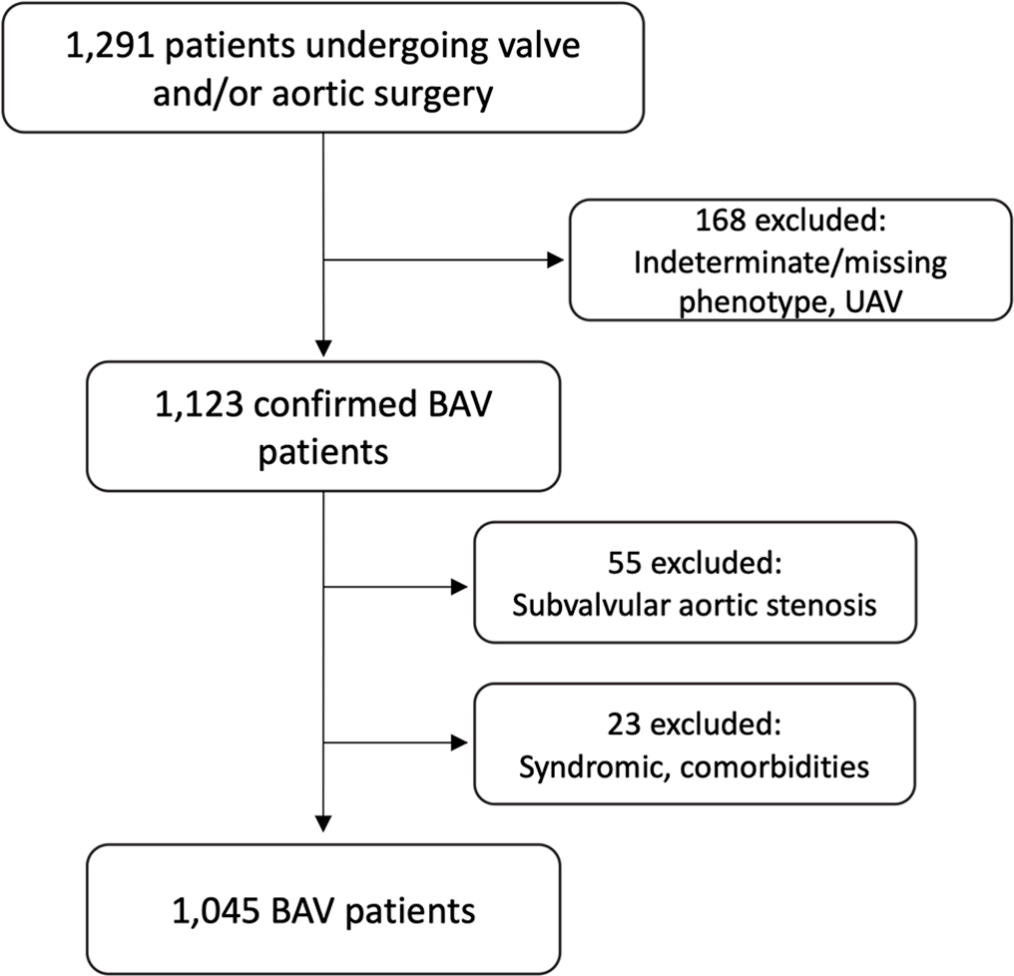
Flow chart of patient selection. BAV, bicuspid aortic valve; UAV, unicuspid aortic valve.

Out of the 1045 included patients (figure 1), there were 794 (76.0%) men and 251 (24.0%) women. Patient demographics, clinical data and echocardiography data are shown in table 1. Female patients were significantly older than male patients in the cohort (p<0.001). In line with the previously published data, RL was the most common phenotype (73.6%), followed by RN (16.2%), 2-sinus BAV (9.2%) and LN (1.1%), with no difference in phenotype distribution pattern between men and women (p=0.634). Due to the low number of female patients with LN (n=2), this phenotype was subsequently excluded from subgroup analysis in women. AS was more common among women than men (p<0.001), whereas isolated AI was more common among men (p<0.001). The aortic valve annulus and all aortic segments were larger in men than in women, and all segments but the ascending aorta remained significantly larger after correction for age and BSA. Aortic dilatation ≥4.5 cm at any segment was more common in men than women (p=0.007), and the root extended aortopathy phenotype was more common in men than women (p<0.001).

**Table 1:** Patient characteristics

	All (n=1045)	Men (n=794)	Women (n=251)	P value
Age, years	56.4±13.5	55.1±13.9	60.3±11.5	<0.001
BSA, m^2^	1.99±0.22	2.05±0.19	1.81±0.20	<0.001
Hypertension, n (%)	438 (41.9)	330 (41.6)	108 (43.0)	0.682
Diabetes, n (%; n=583)	52 (8.9)	36 (8.5)	16 (10.1)	0.553
Dyslipidaemia, n (%)	133 (12.7)	100 (12.6)	33 (13.1)	0.819
CABG, n (%)	51 (4.9)	47 (5.9)	4 (1.6)	0.006
Phenotype				
RL, n (%)	769 (73.6)	590 (74.3)	179 (71.3)	0.634
RN, n (%)	169 (16.2)	122 (15.4)	47 (18.7)	
LN, n (%)	11 (1.1)	9 (1.1)	2 (0.8)	
2-sinus, n (%)	96 (9.2)	73 (9.2)	23 (9.2)	
Valve disease				
Aortic valve stenosis, n (%)	696 (66.6)	491 (61.8)	205 (81.7)	<0.001
Aortic valve insufficiency, n (%)	276 (26.4)	252 (31.7)	24 (9.6)	<0.001
Dilatation only, n (%)	73 (7.0)	51 (6.4)	22 (8.8)	0.205
Aortic dimensions*				
Annulus, cm	2.6±0.4 (n=768)	2.7±0.4 (n=574)	2.3±0.3 (n=194)	<0.001
Sinus of Valsalva, cm	3.7±0.6 (n=887)	3.8±0.6 (n=666)	3.3±0.6 (n=221)	<0.001
Sinotubular junction, cm	3.1±0.6 (n=660)	3.3±0.6 (n=492)	2.8±0.6 (n=168)	<0.001
Ascending aorta, cm	4.2±0.8 (n=919)	4.2±0.8 (n=694)	4.0±0.8 (n=225)	0.581
Aortopathy				
Any dilatation, n (%; n=1003)	351 (35.0)	284 (37.3)	67 (27.8)	0.007
Ascending phenotype, n (%)	271 (27.0)	208 (27.3)	63 (26.1)	0.725
Root phenotype, n (%)	19 (1.9)	18 (2.4)	1 (0.4)	0.058
Root extended, n (%)	61 (6.1)	58 (7.6)	3 (1.2)	<0.001

*P values adjusted for BSA and age.

BSA, body surface area; CABG, coronary artery bypass grafting; LN, left-non-coronary fusion;; RL, right–left fusion; RN, right-non-coronary fusion.

Surgical indications are shown in online supplemental table 1. Women were more often operated due to AS than men (73.3% vs 47.5%, p<0.001), whereas AI was a more common surgical indication among men (29.1% vs 7.2%, p<0.001). The proportion of patients with aortic dilatation as the primary surgical indication did not differ between male and female patients (9.1% vs 10.0%, p=0.675), but women had a lower maximum aortic diameter than men in this group (median 5.0 vs 5.4, p=0.014). Among patients primarily operated due to AS, women had a higher mean gradient than men (55.4 vs 50.8 mm Hg, mean difference 4.6 mm Hg, 95% CI: 1.8 to 7.4, p=0.001).

10.1136/openhrt-2021-001857.supp1 Supplementary data

### Associations between valve disease and phenotype

There was no clear association between any BAV phenotype and AS, which was the most common valve disease in all phenotypes in both sexes (table 2). However, the prevalence of AI was significantly different between phenotypes in men (p=0.046), with RL and LN having the highest proportions of AI (34.1% and 44.4%, respectively). A significant difference in AI prevalence among the phenotypes was also found among female patients (p=0.017), but instead with a RN being associated with higher prevalence of AI (21.3% vs 7.3% for RL, p<0.05). The proportion of patients with isolated aortic dilatation differed by phenotype among women (p=0.026), specifically between RL and RN (11.2% vs 0.0%, p<0.05), a finding that was not observed among male patients. The two women with LN both had significant AS.

**Table 2:** Phenotype associations in male and female patients

	Men					Women			
	RL (n=590)	RN (n=122)	LN (n=9)	2-sinus (n=73)	P value	RL (n=179)	RN (n=47)	2-sinus (n=23)	P value
Age, years	55.5±13.8	55.7±13.5	51.8±20.2	51.3±13.7	0.080	61.0±11.4	59.3±12.6	56.0±9.6	0.125
BSA, m^2^	2.06±0.19	2.05±0.19	1.92±0.20	2.07±0.20	0.136	1.82±0.20	1.78±0.20	1.78±0.15	0.434
Hypertension, n (%)	255 (43.2)	48 (39.3)	3 (33.3)	24 (32.9)	0.337	79 (44.1)	17 (36.2)	11 (47.8)	0.547
Diabetes, n (%; n=583)	29 (9.3)	4 (5.4)	1 (20.0)	2 (5.9)	0.400	10 (8.8)	5 (15.6)	1 (8.3)	0.492
Dyslipidaemia, n (%)	72 (12.2)	17 (13.9)	3 (33.3)	8 (11.0)	0.264	22 (12.3)	6 (12.8)	5 (21.7)	0.436
Valve disease									
Aortic valve stenosis, n (%)	351 (59.5)	85 (69.7)	5 (55.6)	50 (68.5)	0.104	146 (81.6)	37 (78.7)	20 (87.0)	0.742
Aortic valve insufficiency, n (%)	201 (34.1)	31 (25.4)	4 (44.4)	16 (21.9)	0.047	13 (7.3)*	10 (21.3)*	1 (4.3)	0.017
Dilatation only, n (%)	38 (6.4)	6 (4.9)	0 (0.0)	7 (9.6)	0.565	20 (11.2)*	0 (0.0)*	2 (8.7)	0.026
Aortic dimensions†									
Annulus, cm	2.7±0.4 (n=421)	2.6±0.3 (n=90)	2.6±0.3 (n=8)	2.7±0.3 (n=55)	0.271	2.3±0.4 (n=133)	2.3±0.3 (n=40)	2.1±0.2 (n=19)	0.060
Sinus of Valsalva, cm	3.8±0.6 (n=495)*	3.6±0.5 (n=101)*	3.5 ±+0.3 (n=8)	3.7±0.6 (n=62)	0.002	3.3±0.6 (n=156)*	3.2±0.5 (n=41)	2.9±0.5 (n=22)*	0.024
Sinotubular junction, cm	3.3±0.6 (n=412)*	3.1±0.5 (n=87)*	3.1±0.5 (n=7)	3.2±0.5 (n=55)	0.048	2.9±0.6 (n=137)	2.7±0.4 (n=36)	2.7±0.5 (n=19)	0.409
Ascending aorta, cm	4.2±0.9 (n=515)	4.0±0.7 (n=107)	3.8±0.7 (n=8)	4.3±0.7 (n=64)	0.116	4.1±0.8 (n=159)	3.9±0.8 (n=43)	3.7±0.8 (n=21)	0.089
Aortopathy									
Any dilatation, n (%; n=1003)	222 (39.2)	33 (28.7)	1 (11.1)	28 (39.4)	0.065	52 (30.2)	9 (20.5)	5 (21.7)	0.347
Ascending phenotype, n (%)	160 (28.2)	27 (23.5)	1 (11.1)	20 (28.2)	0.569	48 (27.9)	9 (20.5)	5 (21.7)	0.536
Root phenotype, n (%)	13 (2.3)	0 (0.0)*	0 (0.0)	5 (7.0)	0.031	1 (0.6)	0 (0.0)	0 (0.0)	1.000
Root extended, n (%)	49 (8.6)	6 (5.2)	0 (0.0)	3 (4.2)	0.433	3 (1.7)	0 (0.0)	0 (0.0)	1.000

*Groups are significantly different after Bonferroni corrections for multiple comparisons.

†P values adjusted for age and BSA.

BSA, body surface area; LN, left-non-coronary fusion;; RL, right–left fusion; RN, right-non-coronary fusion.

### Aortic dimensions in BAV phenotypes

Men with RL had larger sinus of Valsalva (p=0.002) and sinotubular junction dimensions (p=0.031) than men with other phenotypes, with the largest difference observed between RL and RN at the sinus of Valsalva after adjustment for age and BSA (mean adjusted difference: 0.24 cm, 95% CI :0.06 to 0.41, p=0.002). There was a trend towards different prevalence of aortopathy between phenotypes in men (p=0.063), with RL and 2-sinus BAV having the highest prevalence of dilatation at any segment (39.2% and 39.4%, respectively), and RN having the lowest prevalence (28.7%). This trend remained in a multivariate model, including age, BSA and valve disease (AS or AI) as covariates (OR: 0.643 compared with RL, 95% CI: 0.401 to 1.031, p=0.067, online supplemental table 2). Moreover, 2-sinus BAV was associated with the highest proportion of root phenotype dilatation in men (10.9%, p=0.004), and was independently associated with the root phenotype in a multivariate model (OR: 3.179 compared with RL, 95% CI: 1.065 to 9.489, p=0.038, online supplemental table 3). In contrast, women with 2-sinus BAV did not have root dilatation, and even had significantly smaller sinus of Valsalva dimensions than other phenotypes (mean adjusted difference: 0.35 cm compared with RL, 95% CI: 0.04 to 0.66, p=0.021, adjusted for age and BSA). There was also a trend towards smaller annulus dimensions in women with 2-sinus BAV (p=0.060, adjusted for age and BSA). No significant association between phenotype and aortopathy was found in women. Only one (11.1%) of nine male patients with LN had aortopathy, while one out of two female patients had aortic dilatation, which was limited to the ascending aorta in both cases. No patient of either sex had RN and root phenotype aortic dilatation.

### Type of valve disease and aortic dimensions

Patients with AI were significantly younger than patients with AS in both sexes (p<0.001) (table 3). All aortic root segments were significantly larger in male patients with AI than in male patients with AS, with the largest difference observed at the sinus of Valsalva (mean adjusted difference: 0.40 cm, 95% CI: 0.30 to 0.51, p<0.001, corrected for age and BSA). There was a trend towards larger ascending aorta associated with AI, after correction for age and BSA (p=0.082). The differences in aortic dimensions between AS and AI were even more pronounced in female patients, with the largest difference observed at the level of the sinus (mean adjusted difference: 0.78 cm, 95% CI: 0.55 to 1.02, p<0.001, adjusted for age and BSA). Female patients with AI also had significantly larger ascending aortas than female patients with AS (mean adjusted difference: 0.61 cm, 95% CI: 0.25 to 0.97, p=0.001). The root extended aortopathy phenotype was significantly more common among men with AI compared with AS (11.2% vs 4.1%, p<0.001), but the aortopathy prevalence and pattern did not differ otherwise among male patients. Women with AS did not have root or root extended phenotype aortic dilatation, and it was uncommon among women with AI. However, isolated ascending aortic dilatation was significantly more common among women with AI compared with AS (50.0% vs 17.4%, p<0.001).

**Table 3:** Aortic dimensions and aortopathy by valve disease

Men	Stenosis (n=491)	Insufficiency (n=252)	P value
Age, years	60.1±11.9	45.2±12.6	<0.001
BSA, m^2^	2.05±0.19	2.05±0.19	0.978
Aortic dimensions*			
Annulus, cm	2.6±0.4 (n=345)	2.9±0.4 (n=191)	<0.001
Sinus of Valsalva, cm	3.7±0.5 (n=405)	4.0±0.7 (n=218)	<0.001
Sinotubular junction, cm	3.1±0.5 (n=345)	3.4±0.7 (n=182)	<0.001
Ascending aorta, cm	4.1±0.8 (n=426)	4.2±0.9 (n=220)	0.082
Aortopathy			
Any dilatation, n (%; n=711)	152 (32.4)	84 (34.6)	0.562
Ascending phenotype, n (%)	124 (26.4)	52 (21.4)	0.139
Root phenotype, n (%)	9 (1.9)	5 (2.1)	1.000
Root extended, n (%)	19 (4.1)	27 (11.2)	<0.001
**Women**	**Stenosis (n=205**)	**Insufficiency (n=24**)	**P value**
Age, years	62.0±10.8	52.2±13.1	<0.001
BSA, m^2^	1.80±0.20	1.85±0.17	0.223
Aortic dimensions*			
Annulus, cm	2.2±0.3 (n=158)	2.5±0.3 (n=20)	<0.001
Sinus of Valsalva, cm	3.1±0.4 (n=178)	3.8±1.0 (n=23)	<0.001
Sinotubular junction, cm	2.7±0.4 (n=156)	3.3±1.1 (n=20)	<0.001
Ascending aorta, cm	3.8±0.8 (n=181)	4.4±0.6 (n=22)	0.001
Aortopathy			
Any dilatation, n (%; n=219)	34 (17.4)	14 (58.3)	<0.001
Ascending phenotype, n (%)	34 (17.4)	12 (50.0)	<0.001
Root phenotype, n (%)	0 (0.0)	1 (4.2)	0.110
Root extended, n (%)	0 (0.0)	1 (4.2)	0.110

*P values adjusted for age and BSA.

BSA, body surface area.

## Discussion

The present surgical cohort study describes, for the first time, sex-dependent associations between BAV phenotypes, type of valve disease, aortic dimensions and aortopathy. We found that AI was most common with RL and LN in male patients, whereas AI was significantly more common with RN in female patients. The 2-sinus phenotype was associated with a high prevalence of root phenotype aortic dilatation in men. In contrast, women with 2-sinus BAV did not have root dilatation and had smaller sinus dimensions. Further, the difference in aortic dimensions and aortopathy prevalence between AS and AI was more pronounced in female patients.

One of the most notable sex differences in the present study was the strong correlation between the RN phenotype and AI in female patients. In contrast, earlier studies in mixed-gender non-surgical cohorts have reported a higher prevalence of AS with RN or no difference between phenotypes.^13 23^ RN has also been associated with a larger ascending aorta in both adult and paediatric cohorts,^23 24^ but this relationship has not been consistently demonstrated.^13–15 25^ On the other hand, our results indicate that RN could be associated with slightly lower risk of aortic dilatation, at least in male patients, and the root phenotype was not seen in any patient with RN, regardless of sex. We also found that male patients with 2-sinus BAV had a high prevalence of root dilatation independent of the type of valve disease, consistent with previous findings in a surgical series,^25^ whereas female patients with 2-sinus BAV did not have root dilatation and even had smaller root dimensions than other phenotypes.

We could confirm the previously established correlation between the RL phenotype and larger aortic root dimensions, which is well-documented in echocardiography cohorts.^11–14^ The relationship was most evident in male patients, in which RL was also associated with a high prevalence of AI. Non-surgical cohorts have previously shown similar AI rates between phenotypes.^23^ Further, patients with 2-sinus BAV tend to present clinically at an earlier age than patients with other phenotypes,^15 18^ and a similar trend could be seen in men in the present study.

AI has been shown to correlate with larger aortic dimensions, and with root and root extended phenotype aortic dilatation, whereas AS has been associated with ascending phenotype dilatation.^11 23 25 26^ In the present study, almost all aortic dimensions were larger in AI in both sexes, but the difference in dimensions was greater in women. Only the root extended phenotype of dilatation was more common with AI in men, whereas ascending dilatation was significantly more common in women with AI compared with AS.

General sex differences in BAV cohorts have previously been reported by others. We could confirm that women tend to be older at the time of surgery and more often have AS, whereas men are younger and more often have AI.^3 11 13 19 20^ Similar to the results of Andrei *et al*^20^, the female patients in the present cohort had more severe AS at the time of surgery, which implies that women with AS may present and/or be referred for surgery with more advanced stenosis than men.

Our study has some limitations. Due to selection bias, it was not possible to compare aortic dimensions between patients with diseased and normally functioning valves, nor could we assess differences related to the degree of valve disease. The study also lacks longitudinal data, which are necessary to confirm the findings. However, it is one of the largest surgical cohorts to date with phenotype data, which provide verification of BAV diagnosis and phenotype classification. The lower number of female patients still means that some associations seen in male patients may have been statistically non-significant in female patients due to fewer cases. The study could nevertheless demonstrate phenotype associations uniquely found in women. The overall size of the study also allows the inclusion of more uncommon phenotypes, in particular 2-sinus BAV, which is commonly excluded due to its relative scarcity.^11 13 14 23^

## Conclusions

The present study describes, for the first time, sex-dependent associations between BAV phenotypes, valve disease, aortic dimensions and aortopathy. Specifically, RL was associated with high prevalence of AI in male patients, whereas AI was significantly more common in RN in female patients. The 2-sinus phenotype was associated with a high prevalence of root phenotype aortic dilatation in men independently of the type of valve disease, while women with 2-sinus BAV did not have root dilatation and even had smaller sinus dimensions than other phenotypes. BAV phenotype associations found in cohorts with a male predominance can thus not be extrapolated to female patients. Differences in aortic dimensions and aortopathy prevalence between AS and AI were larger in women. We could also confirm some earlier findings, such as the association between RL and larger sinus dimensions, seen in male patients, and that women with BAV and AS may be diagnosed or referred for surgery with more severe stenosis than men with BAV. Although longitudinal studies are necessary to determine the clinical implications, these findings provide important insights into sex differences in the associations between BAV phenotype, type of valve disease, aortic dimensions and aortopathy pattern, which may be important factors in individualised decision-making for patients with BAV.

## Data Availability

All data relevant to the study are included in the article or uploaded as supplementary information. Deidentified participant data.
