# Supplementary material for: Valve disease and aortopathy associations of bicuspid aortic valve phenotypes differ between men and women
Source: Open Heart. 2021 Oct 20;8(2):e001857. doi: 10.1136/openhrt-2021-001857 (PMC8529975; doi:10.1136/openhrt-2021-001857)
Supplement: Supplementary data [file openhrt-2021-001857supp001.pdf]

Supplemental Data

Supplemental Table 1. Surgical indications.

SURGICAL INDICATION	All (n=1045)	Men (n=794)	Women (n=251)	P-value
Aortic valve stenosis, n (%)	561 (53.7)	377 (47.5)	184 (73.3)	<0.001
Mean gradient, mmHg	52.3 ± 15.0 (n=539)	50.8 ± 14.0 (n=363)	55.4 ± 16.4 (n=176)	0.001
Severe AS, n (%)	479 (45.9)	315 (39.7)	164 (65.3)	<0.001
Moderate AS with dilatation ≥4.5 cm, n (%)	51 (4.9)	43 (5.4)	8 (3.2)	0.153
Severe AS with dilatation ≥5.5 cm, n (%)	12 (1.1)	7 (0.9)	5 (2.0)	0.173
Aortic valve insufficiency, n (%)	249 (23.8)	231 (29.1)	18 (7.2)	<0.001
Severe AI, n (%)	202 (19.3)	187 (23.6)	15 (6.0)	<0.001
Moderate AI with dilatation ≥4.5 cm, n (%)	34 (3.3)	27 (3.4)	7 (2.8)	0.634
Severe AI with dilatation ≥5.5 cm, n (%)	14 (1.3)	13 (1.6)	1 (0.4)	0.208
Aortic dilatation, n (%)	97 (9.3)	72 (9.1)	25 (10.0)	0.671
Maximum diameter, cm, median [IQR]	5.3 [0.8]	5.4 [0.7]	5.0 [0.8]	0.014
Aorta ≥5.5 cm, n (%)	36 (3.4)	31 (3.9)	5 (2.0)	0.148
Other cardiac surgery, n (%)	15 (1.4)	13 (1.6)	2 (0.8)	0.542
AS or AI, degree missing, n (%)	8 (0.8)	8 (1.0)	0 (0.0)	0.210
Aortic dilatation, dimensions missing, n (%)	1 (0.1)	1 (0.1)	0 (0.0)	1.000
AS or AI and aortic dilatation, severity and dimensions missing, n (%)	3 (0.3)	2 (0.3)	1 (0.4)	0.562

AS=aortic valve stenosis. AI=aortic valve insufficiency. IQR=interquartile range.

Supplemental Table 2. Correlations with any aortic dilatation, male patients (n=712).

UNIVARIATE	**OR**	**95% CI**	**P-value**
Age	1.004	0.993 - 1.015	0.499
BSA	5.44	2.322 - 12.749	<0.001
Dyslipidemia	0.916	0.568 - 1.476	0.718
Hypertension	1.176	0.857 - 1.613	0.315
CABG	1.129	0.588 - 2.165	0.716
**Valve disease (Ref: AS)**			
AI	1.102	0.794 - 1.529	0.562
**Phenotype (Ref: RL)**			
RN	0.639	0.401 - 1.018	0.059
LN	0.231	0.029 - 1.863	0.169
2-sinus	0.903	0.52 - 1.568	0.718
MULTIVARIATE			
Age	1.01	0.996 - 1.023	0.155
BSA	5.775	2.425 - 13.753	<0.001
**Valve disease (Ref: AS)**			
AI	1.24	0.839 - 1.833	0.281
**Phenotype (Ref: RL)**			0.205
RN	0.643	0.401 - 1.031	0.067
LN	0.287	0.035 - 2.355	0.245
2-sinus	0.916	0.521 - 1.61	0.761

BSA=body surface area. CABG=coronary artery bypass grafting. AS=aortic valve stenosis. AI=aortic valve insufficiency. RL=right-left fusion. RN=right-non-coronary fusion. LN=left-non-coronary fusion. OR=odds ratio.

Supplemental Table 3. Correlations with root phenotype dilatation, male patients (n=712).

UNIVARIATE	OR	95% CI	P-value
Age	0.987	0.951 - 1.024	0.479
BSA	0.557	0.031 - 10.095	0.692
Hypertension	1.080	0.371 - 3.147	0.887
Dyslipidemia	0.000	-	0.997
CABG	1.233	0.157 - 9.655	0.842
**Valve disease (Ref: AS)**			
AI	1.074	0.356 - 3.240	0.899
**Phenotype (Ref: RL)**			
RN	0.000	-	0.996
LN	0.000	-	0.999
2-sinus	4.906	1.591 -15.124	0.006
MULTIVARIATE			
Age	0.999	0.960 - 1.039	0.947
BSA	0.522	0.039 - 6.965	0.623
**Valve disease (Ref: AS)**			
AI	0.842	0.250 - 2.835	0.781
**Phenotype (Ref: RL)**			
RN	0.000	-	0.996
LN	0.000	-	0.999
2-sinus	4.810	1.516 - 15.259	0.008

BSA=body surface area. CABG=coronary artery bypass grafting. AS=aortic valve stenosis. AI=aortic valve insufficiency. RL=right-left fusion. RN=right-non-coronary fusion. LN=left-non-coronary fusion. OR=odds ratio.
